# Anatomical Measurements of the Malar Bone for Safe Zygomatic Implant Placement: A Study on Donated Bodies

**DOI:** 10.3390/jcm13226798

**Published:** 2024-11-12

**Authors:** Carlo Barausse, Pietro Felice, Roberto Pistilli, Gerardo Pellegrino, Lorenzo Bonifazi, Subhi Tayeb, Antonietta Fazio, Maria Vittoria Marvi, Lucia Manzoli, Stefano Ratti

**Affiliations:** 1 Cellular Signaling Laboratory, Anatomy Center, Department of Biomedical and Neuromotor Sciences (DIBINEM), University of Bologna, 40126 Bologna, Italy; antonietta.fazio2@unibo.it (A.F.); mariavittoria.marvi2@unibo.it (M.V.M.); lucia.manzoli@unibo.it (L.M.); stefano.ratti@unibo.it (S.R.); 2 Oral Surgery Unit, Department of Biomedical and Neuromotor Sciences (DIBINEM), University of Bologna, 40125 Bologna, Italy; pietro.felice@unibo.it (P.F.); gerardo.pellegrino2@unibo.it (G.P.); lorenzo.bonifazi@unibo.it (L.B.); subhi.tayeb@studio.unibo.it (S.T.); 3 Oral and Maxillofacial Unit, San Camillo Hospital, 00152 Rome, Italy; roberto.pistilli@unibo.it

**Keywords:** zygoma, zygomatic implants, anatomy, intraoperative complications

## Abstract

**Background**: The malar bone provides an anchorage point for zygomatic implants, avoiding invasive reconstructive surgeries in the fixed rehabilitation of fully edentulous and severely atrophic maxillae. The limited bone volume, however, requires precise implant placement to prevent complications related to nearby anatomical structures. This observational cross-sectional study aims to measure the malar and zygomatic arch bones and their distances from critical anatomical landmarks to guide surgeons in safe zygomatic implant placement. **Methods**: Dissections were performed bilaterally on 29 heads from human donated bodies in a cross-sectional observational study. Key landmarks evaluated include the infraorbital foramen (IF), pyriform nasal aperture (PNA), infraorbital margin (IM), zygomaticofacial foramen (ZFF), anterior end (A), and the most protruding point of the zygomatic arch (B). Measurements included IF-PNA, IF-IM, IF-ZFF, ZFF-IM, A-B, and orbital floor depth (OFD). **Results**: Significant findings showed IF-PNA was greater in males (18.66 ± 2.63 mm, *p* = 0.001), and IF-ZFF varied between sides (26.72 ± 8.7 mm, *p* = 0.002). ZFF-IM was larger in males (7.43 ± 2.09 mm, *p* < 0.001). Heights and thicknesses were also assessed, with significant side differences observed. **Conclusions**: These findings underscore the importance of understanding precise anatomical distances for successful implant placement. The study provides essential data to enhance surgical planning and training, ensuring safer procedures and minimizing the risk of complications.

## 1. Introduction

Nowadays, edentulism affects approximately 158 million people worldwide (2.3% of the population) and poses a significant public health concern due to its high prevalence, exceeding 10% in adults over 50 years of age [1,2]. Dental implants are the gold standard treatment option for edentulous patients. However, severe bone resorption precludes their placement, and this is even more common today due to the increase in the average patient age [3].

One solution could be to reconstruct the bone; however, in such severe cases this can be unpredictable and associated with complications [4].

For fully edentulous, severely atrophic patients, particularly those with previous reconstructive surgery failures, zygomatic implants have emerged as a viable treatment option. These longer implants utilize the zygomatic bone as anchorage, allowing an immediate fixed implant-supported rehabilitation [5,6]. Nevertheless, their placement demands a highly skilled and experienced surgeon capable of navigating the complex surgical anatomy. While zygomatic implants offer a valuable solution in challenging cases, it is important to acknowledge that their placement is not without risks due to their proximity to critical anatomical structures. In the literature, nerve paresthesia, accidental penetration into the infratemporal fossa and orbital cavity during surgery, and even intracerebral penetration were reported [7,8,9,10,11,12,13,14,15].

Mitigating these complications is possible only with a thorough understanding of the anatomy. To fulfill this goal, different models can be involved, both artificial and biologic; however, donated bodies remain the most effective for faithfully reproducing complex anatomical scenarios. Indeed, while the use of artificial models can still be effective and beneficial—especially in undergraduate courses, where exercises are generally less complex—for postgraduate training courses and for surgical research purposes, the resort to dissecting donated human bodies becomes inevitable [16].

The present study aims to measure the malar and zygomatic arch bones and distances to the relevant anatomical structures, to guide the surgeon in a correct and safe zygomatic implant insertion. By analyzing key anatomical landmarks and dimensions in human donated bodies, this research aims to contribute to a more precise and intraoperative complication-free surgical approach for zygomatic implant placement.

## 2. Materials and Methods

An observational cross-sectional study was conducted. The present study received approval from the University of Bologna School of Medicine bioethical board, Italy (Prot. N. 0102300 of 10 April 2024). The bodies donated to science used in this study are part of the Italian National Program for Body Donation to Science (Law N. 10 of February 2020). The donors provided consent for research and training activities, as well as for the acquisition and preservation of images and video recordings. The body donors were treated with the utmost respect and in accordance with the recent guidelines issued by the International Federation of Associations of Anatomists. The study was conducted in agreement with EU-GDPR and the Helsinki Declaration.

### 2.1. Anatomical Landmarks

**Figure 1:**
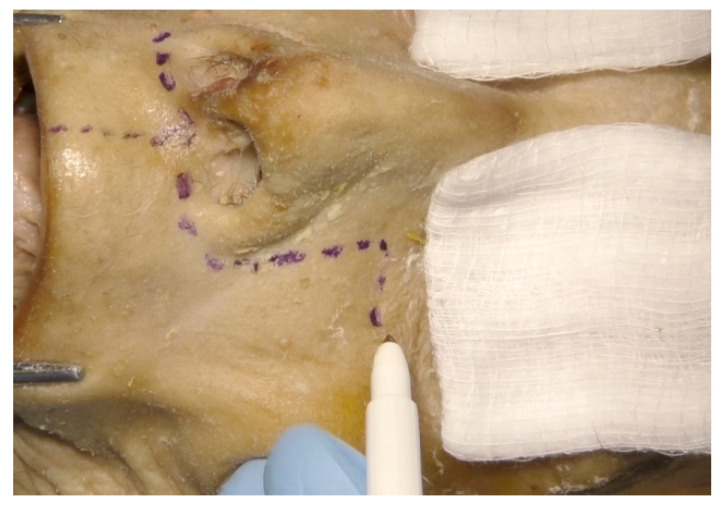
Weber–Ferguson incision used to allow access to the maxilla and the zygomatic bone and arch.

The heads were bilaterally dissected with a Weber–Ferguson approach, which is a surgical technique involving an incision from the upper lip, around the nose, to the lateral eye, allowing access to the maxilla and the zygomatic bone and arch [17] (Figure 1). 

**Figure 2:**
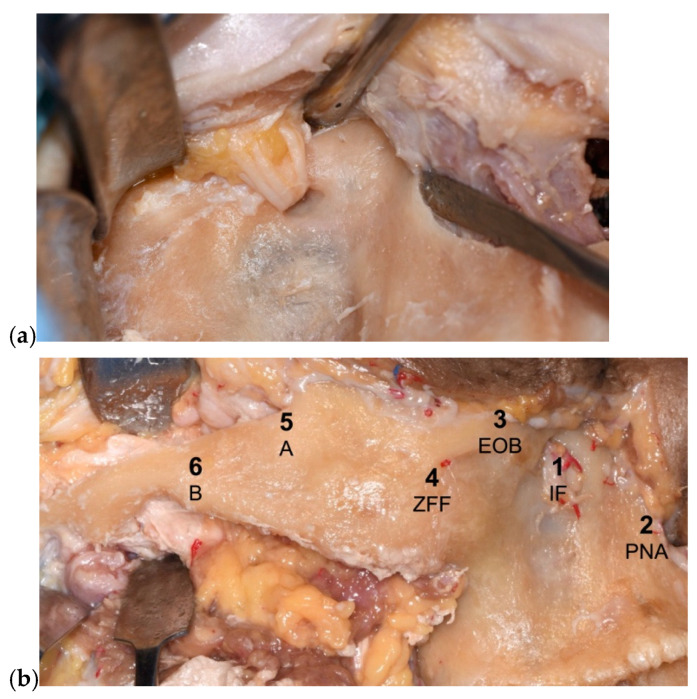
Anatomical landmarks involved in zygomatic surgery that were taken into consideration: (**a**) infraorbital nerve, (**b**) overview of all the considered points [infraorbital foramen (IF), pyriform nasal aperture (PNA), external orbital border (EOB) or infraorbital margin, zygomaticofacial foramen (ZFF), anterior end of the zygomatic arch (A), most protruding point of the zygomatic arch (B)].

The main anatomical landmarks involved in the zygomatic surgery that were taken into consideration were the following: the infraorbital foramen (IF), the pyriform nasal aperture (PNA), the infraorbital margin (IM), the zygomaticofacial foramen (ZFF), the anterior end of the zygomatic arch (A), and the most protruding point of the zygomatic arch (B). The presence/absence and the number of ZFFs were assessed and recorded for each malar bone (Figure 2).

**Figure 3:**
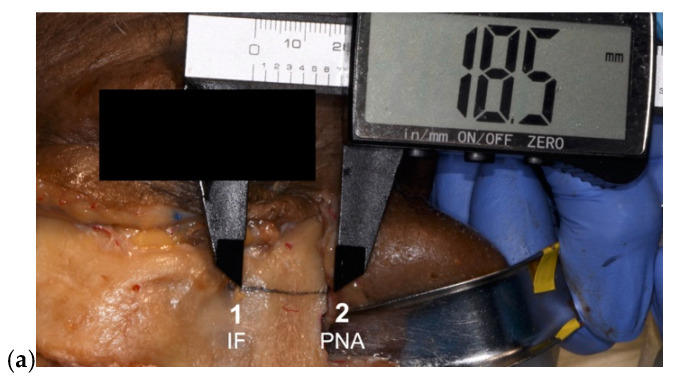

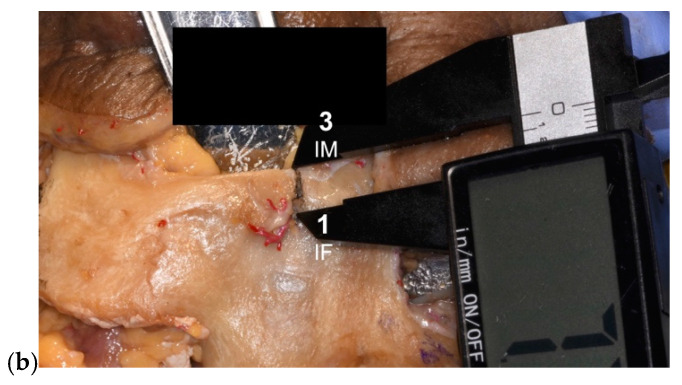
(**a**,**b**) Examples of linear measurements taken with a digital caliper with a resolution of 0.1 mm [infraorbital foramen (IF), pyriform nasal aperture (PNA), infraorbital margin (IM)].

The following linear measurements were taken with a digital caliper (resolution 0.1 mm) from each zygoma: the distances IF-PNA, IF-IM, IF-ZFF, ZFF-IM, and A-B. The vertical orbital floor depth (OFD) was measured from the central portion of the infraorbital margin with a probe (resolution 1 mm), using the orbital rim as a reference (Figure 3).

**Figure 4:**
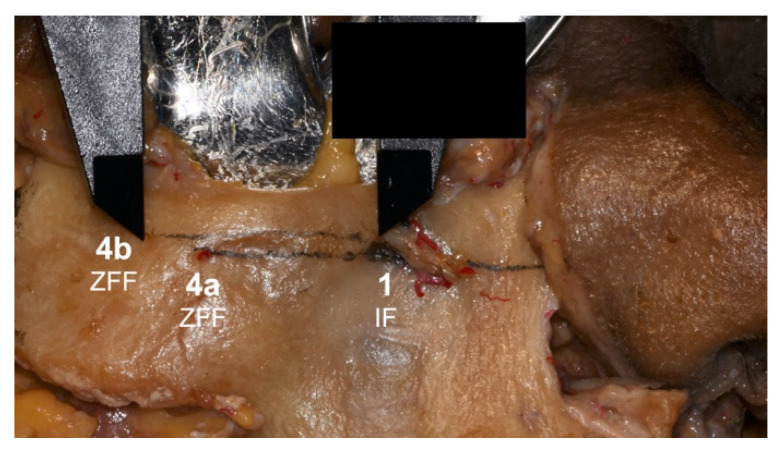
Linear distances between the infraorbital foramen (IF) and the two zygomaticofacial foramens (ZFFs).

In cases where more than one ZFF was found in the same side, the linear measurements were averaged for each side (Figure 4).

Malar and zygomatic arch height and thickness were taken with A and B as reference points. Sex, age, ethnicity, and type of edentulism (partial or total) were also considered in the analysis.

These specific anatomical landmarks were selected due to their critical relevance in zygomatic implant placement. The infraorbital foramen (IF) was included because it transmits the infraorbital nerve and vessels; measuring distances from the IF is essential to avoid nerve injury during drilling, which can lead to midfacial paresthesia. The pyriform nasal aperture (PNA) serves as a reference point for the anterior maxilla, aiding in vertical implant positioning and preventing encroachment into the nasal cavity. The infraorbital margin (IM), being the inferior rim of the orbit, is crucial for preventing orbital penetration during implant placement; measurements involving the IM help avoid ocular complications. The zygomaticofacial foramen (ZFF) transmits the zygomaticofacial nerve; identifying its position, when its caliber is relevant, is important to prevent nerve damage and subsequent sensory disturbances on the lateral aspect of the face. Point A, the anterior end of the zygomatic arch, marks the beginning of the arch. Measuring at this point provides information on the bone height and thickness available for possible implant anchorage in extremely atrophic patients. This is particularly important for determining the appropriate implant dimensions and ensuring sufficient bone support. Point B, the most protruding point of the zygomatic arch, represents the area of maximal lateral projection. As Point B usually has reduced height and thickness, we aimed to further show how this area is not suitable to be reached with a zygomatic implant placement. The orbital floor depth (OFD) measurement indicates the vertical depth of the orbital floor from the infraorbital margin. This measurement is essential to avoid breaching the orbital cavity during drilling, thus preventing severe complications such as ocular damage.

### 2.2. Statistical Analysis

Descriptive statistics were computed for continuous variables, including means, standard deviations, and maximum and minimum values at both the head level and the measurement level. Categorical variables were analyzed in terms of both percentages and absolute values. The normality of the data was assessed using the Shapiro–Wilk and Kolmogorov–Smirnov tests and graphical methods. Statistical analysis was further extended to each linear measurement, investigating factors such as age, sex, type of edentulism (partial or total), and side (left or right). Each model employed mixed effects with REML (Restricted Maximum Likelihood) estimation to accommodate data clustering. The significance level was set at 0.05. The analyses were conducted using Stata 18 software (StataCorp. 2023 Release 18. StataCorp LLC: College Station, TX, USA).

## 3. Results

### 3.1. Demographic Data

This study involved measurements taken from 29 heads of donated full bodies. The average age of the sample was 69.79 years. Regarding sex distribution, the sample consisted of 19 males, accounting for 65.52% of the total, and 10 females, representing 34.48% of the total. All individuals in the sample were of Caucasian ethnicity. In terms of dentition status, 19 individuals (65.52%) were partially edentulous, while 10 individuals (34.48%) were totally edentulous.

### 3.2. Linear Measurements and Observations

**Scheme 1:**
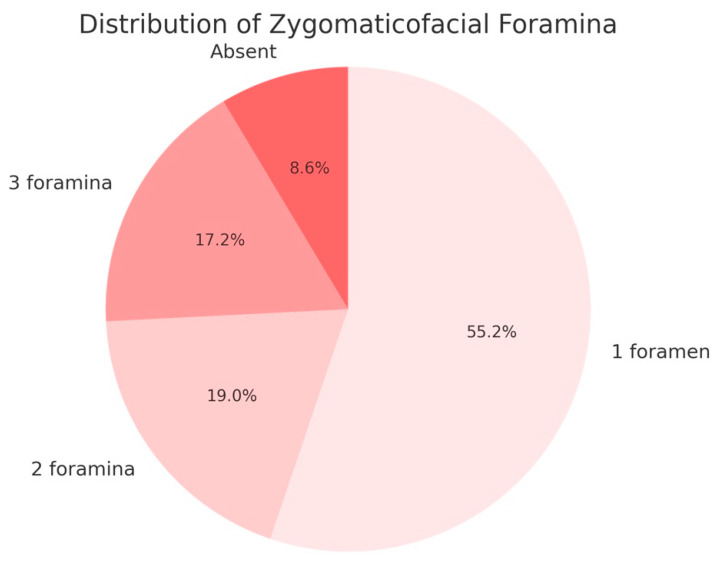
Distribution of zygomaticofacial foramina in the studied sample.

For each head, measurements were taken from both the left and right sides, totaling 29 measurements from each side. The presence of the zygomaticofacial foramen (ZFF) varied as follows: 32 sides (55.17%) had one foramen, 11 sides (18.97%) had two foramina, 10 sides (17.24%) had no foramen, and 5 sides (8.62%) had three foramina (Scheme 1).

**Scheme 2:**
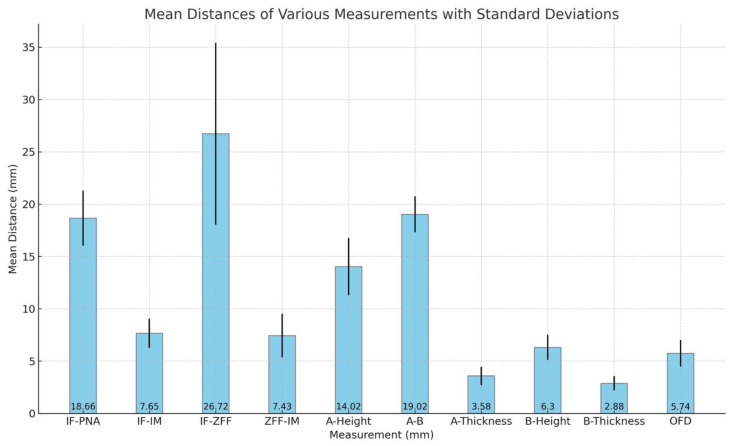
Mean distances (mm) with standard deviations for various anatomical measurements related to the malar bone and zygomatic arch. The measurements include infraorbital foramen to pyriform nasal aperture (IF-PNA), infraorbital foramen to orbital floor (IF-OF), infraorbital foramen to zygomaticofacial foramen (IF-ZFF), zygomaticofacial foramen to orbital floor (ZFF-OF), height at point A (A-Height), distance from point A to point B (A-B), thickness at point A (A-Thickness), height at point B (B-Height), thickness at point B (B-Thickness), and orbital floor depth (OFD).

The infraorbital foramen to pyriform nasal aperture (IF-PNA) distance was 18.66 ± 2.63 mm. The infraorbital foramen to infraorbital margin (IF-IM) distance measured 7.65 ± 1.39 mm. The infraorbital foramen to zygomaticofacial foramen (IF-ZFF) distance was 26.72 ± 8.7 mm, while the zygomaticofacial foramen to infraorbital margin (ZFF-IM) distance was 7.43 ± 2.09 mm. The height related to point A (the anterior end of the zygomatic arch) was 14.02 ± 2.74 mm. The thickness related to point A was 3.58 ± 0.86 mm. The height related to point B (the most protruding point of the zygomatic arch) was 6.3 ± 1.2 mm, and the thickness related to point B was 2.88 ± 0.68 mm. The distance from point A to point B was 19.02 ± 1.72 mm. The depth of the border of the orbital floor (OFD) was 5.74 ± 1.28 mm (Table 1, Scheme 2).

**Scheme 3:**
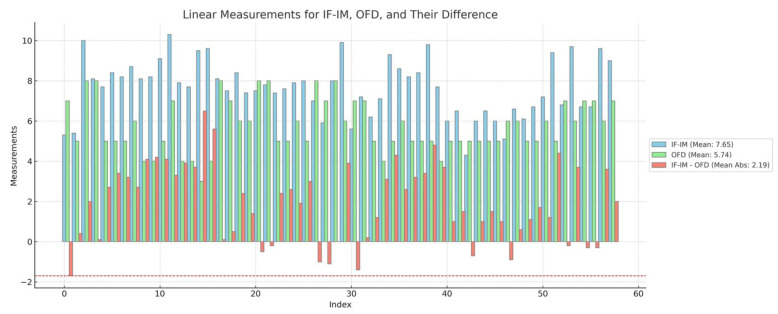
Linear measurements for infraorbital foramen to external border of the orbital floor (IF-OF), orbital floor depth (OFD), and their difference in terms of absolute values. The red line represents the minimum value found in the differences between the two measurements.

Finally, the difference in distance between the infraorbital foramen (IF) and the vertical orbital floor depth (OFD) was calculated, with an average absolute value of 2.2 mm and a minimum value of −1.7 mm, indicating that the orbit can sometimes extend beneath the infraorbital nerve (Scheme 3).

### 3.3. Correlations with Variables

**Scheme 4:**
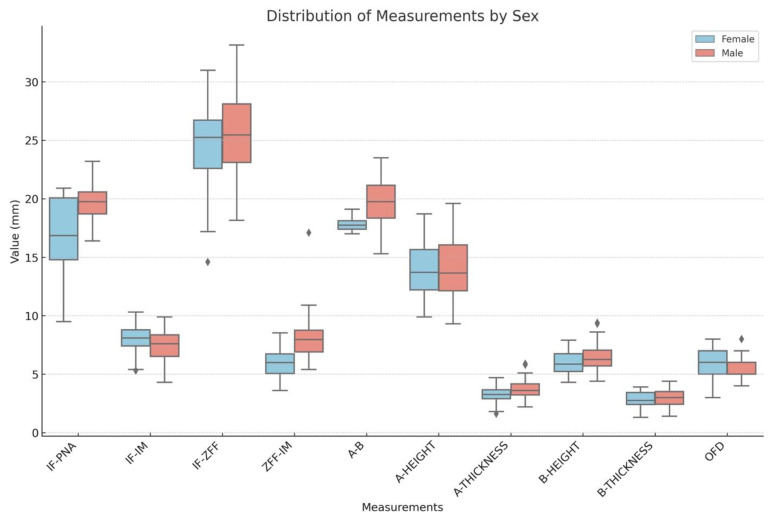
Boxplot for the distribution of measurements based on sex (male and female); (♦: outliers data).

**Scheme 5:**
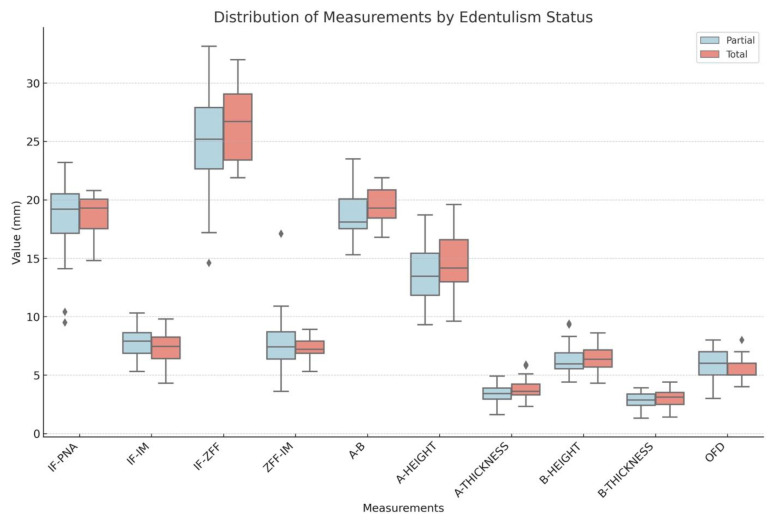
Boxplot for the distribution of measurements based on the type of edentulism (total or partial); (♦: outliers data).

**Scheme 6:**
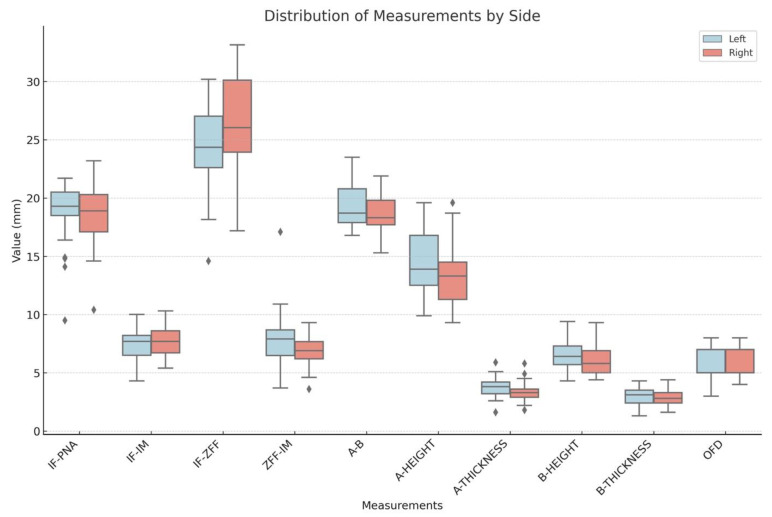
Boxplot for the distribution of measurements based on the side (right and left); (♦: outliers data).

**Scheme 7:**
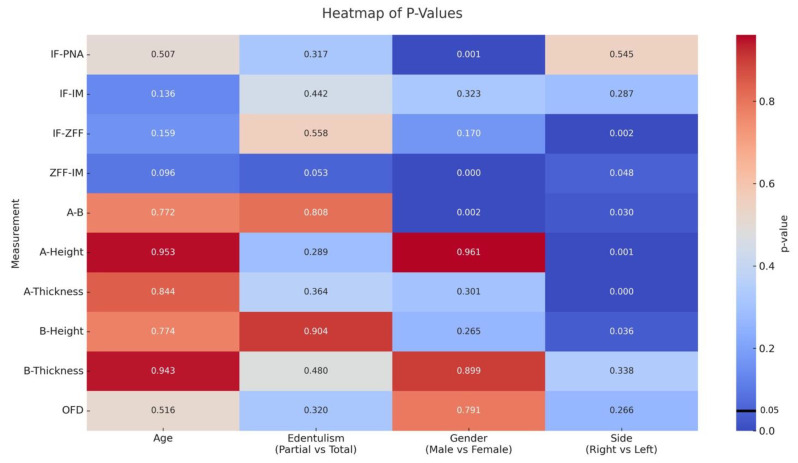
The heatmap visualizes the *p*-values derived from a mixed-effects model using REML estimation for different measurements and variables. Each cell represents the *p*-value of a specific measurement–variable pair. The cells are color-coded using a cool–warm palette, with cooler colors indicating lower *p*-values and warmer colors indicating higher *p*-values.

For the IF-PNA distance, gender showed a significant effect (*p* = 0.001), indicating that males generally had a greater distance compared to females. Age, edentulism, and side of measurement did not show significant effects. For the IF-IM distance, none of the variables (age, gender, edentulism, and side of measurement) showed significant effects, with all *p*-values being above the threshold for significance. The IF-ZFF distance was significantly affected by the side of measurement (*p* = 0.002), indicating that there was a difference between the left and right sides. Gender, age, and edentulism did not have significant effects. For the ZFF-IM distance, males had a significantly larger distance (*p* < 0.001), and differences were observed between sides (*p* = 0.048). Age and edentulism did not significantly influence this measurement. The height related to point A and the thickness related to point A showed significant differences between the right and the left sides (*p* < 0.001). Gender, age, and edentulism were not significant. The height related to point B was significantly affected by the side of measurement (*p* = 0.036). Age, gender, and edentulism did not significantly affect this measurement. Regarding the thickness related to point B, no difference was observed in terms of age, gender, and type of edentulism. In terms of A-B distance, both gender and side of measurement showed significant effects. A significant increase in this distance was found in males (*p* = 0.002), with differences observed between sides (*p* = 0.030). Age and edentulism were not significant factors. Lastly, for the vertical orbital floor depth (OFD), none of the variables (age, gender, edentulism, and side of measurement) showed significant effects, indicating no significant associations (Scheme 4, Scheme 5, Scheme 6 and Scheme 7).

## 4. Discussion

A deep understanding of human anatomy is essential for medical professionals and is a critical milestone both in pregraduate and postgraduate programs, including the study and training phases, as well as for medical research purposes. One effective way to learn and practice is through the dissection of donated human bodies, which still stands as an excellent method to achieve a high-fidelity and realistic three-dimensional comprehension of human anatomy [16,18]. This knowledge is particularly crucial for complex surgical techniques where even minor mistakes can result in intraoperative complications, like zygomatic implant placement. Moreover, in advanced surgical scenarios, new technologies, such as the full-body revascularization and ventilation of donated bodies, should be considered as an important added value [19]. Additionally, the environmentally friendly attributes of using donated human bodies, which already exist in nature, coupled with their educational effectiveness, highlight their value over synthetic models.

### 4.1. Zygomatic Implant Advantages

Zygomatic implants represent a technique suitable for treating severe maxillary bone atrophies where effective bone reconstructions or conventional dental implant placements are not feasible. Fixed rehabilitation with these implants offers several advantages, including reduced rehabilitation times due to immediate loading and rapidly improved patient quality of life [20,21]. Numerous studies report encouraging survival rates for zygomatic implants, exceeding 96% after five years [22,23]. The present study confirms that the malar bone is not prone to resorption after tooth loss, showing no significant differences in measured distances and points between edentulous patients and those with remaining teeth. Therefore, zygomatic implants can be placed even in extremely atrophic patients.

### 4.2. Complications and Safety Considerations

The limited available space and intraoral approach, coupled with the uncommon nature of this rehabilitative technique, underscore the importance of a strong anatomical knowledge of this specific region. In fact, this approach is associated with a high number of complications, the severity of which can be important, particularly for the intraoperative ones [24]. As reported in a Cochrane Database systematic review, orbit penetration can be a severe intraoperative complication, occurring in up to 6% of patients [7]. Penetration into the orbital cavity during the placement of zygomatic implants is a surgical complication that can compromise both visual function and ocular movement [9,10,11,12]. A complication closely linked to the experience of the surgeon is paresthesia of the infraorbital and zygomaticofacial nerve, reported in 5.4% of patients [13]. Extremely rare but dangerous complications include intracerebral penetration, which presents symptoms like severe headache and chronic fatigue, and invasion of the infratemporal fossa, potentially damaging structures like the temporalis muscle and adipose tissue [14,15].

Such biological complications can be prevented, as they are related to surgical mistakes or, more simply, to wrong implant placement in the malar region. This can mainly be blamed on poor anatomical knowledge and limited surgical training on donated bodies, which are crucial for precision surgeries aimed at reducing complications. Although three-dimensional radiology must be considered fundamental in the preoperative study of the individual clinical case, this alone is not sufficient without solid anatomic study and training. Moreover, Computed Tomography (CT) can give the clinicians specific anatomic information of the area to be treated; however, potential distortions or artifacts may reduce the sensitivity of the exam and may not giving the surgeon perfect, trustworthy linear distances from notable anatomical structures [25]. Moreover, it is not always possible to highlight some of them from the CT, particularly in cases of multiple smaller ZFFs. While the zygomaticofacial foramen (ZFF) is a notable anatomical structure, its influence is generally minimal unless there are significant variations. In most clinical scenarios, the presence and variations in the ZFF do not substantially impact the surgical approach, except when dealing with larger calibers or multiple foramina which could complicate the procedure.

### 4.3. Significant Findings and Clinical Implications

In the present study, two major differences were found. First of all, distances such as IF-PNA, ZFF-IM, and A-B distance are influenced by sex, with increased values in men. This highlights the importance of adequate planning and choice of implant dimensions according to the sex of the individual patient and to the intraoperative clinical situation. Moreover, there is apparently no symmetry between sides regarding IF-ZFF, ZFF-IM, and A-B distance, as well as for the height related to point A and the thickness related to point A. This last finding is very important to remember, particularly in cases of anatomical variation, where we cannot take for granted having the same measurements or landmark locations on both sides. This observation aligns with findings from a study on facial asymmetry, which highlights that a certain degree of asymmetry is inherent and can significantly vary among individuals, emphasizing the need for careful individualized assessment [26].

Given the limited bone thickness observed, we recommend the use of zygomatic implants with the smallest possible diameter. Employing implants of smaller diameter can reduce the risk of bone fracture during drilling and implant placement, as well as preserve more of the surrounding bone tissue. This approach accommodates the narrow anatomical spaces and variations identified in our study, enhancing the safety of the procedure.

### 4.4. Safety Line Suggestion

Having a solid knowledge of the anatomical structures and their precise average measurements and distributions in the malar region could be helpful for a safe zygomatic implant surgery. Each clinical case must be precisely evaluated with a CT scan to obtain specific three-dimensional measurements of the area [25,27]. However, these measurements may not be easily applied during surgery due to limited visibility and the patient’s lying position. In this study, the difference in distance between the IF-IM and the OFD was also calculated, with an average absolute value of 2.2 mm. The minimum value of −1.7 mm indicates that the orbit can sometimes extend beneath the infraorbital nerve. This suggests a safety line approximately 4 mm caudal to the IF and through point A, below which zygomatic implants could be safely positioned with a safety margin of 2.3 mm, at least in the studied sample.

The choice of the right implant diameter and length is influenced by the malar bone height and thickness, in order to reduce the risk of bone fracture and to preserve more bone around the fixtures. For the same reason, it is now indicated to use a zygomatic implant with a reduced diameter in the apical portion.

### 4.5. Study Limitations

It is important to consider the limitations of this study, given the relatively small number of human donated bodies involved.

All specimens were of Caucasian ethnicity, which may not represent the anatomical diversity across different populations. Anatomical variations can occur among different ethnic groups, so our findings may not be applicable to all patient populations. Future studies should include a more diverse demographic to determine the consistency of measurements across various ethnicities.

The use of manual measurements with digital calipers and probes introduces the potential for human error and variability. Variations between donated bodies and living patients, such as differences in soft tissues and intraoperative factors, could limit the direct clinical application.

Moreover, a further limitation, which will be explored in future studies, is related to the absence of radiographic tri-dimensional evaluation of the presented measurements.

In the end, it should be remarked that donating the body to science is an ethical act of paramount importance to further increase the chances for students and doctors to deepen their anatomical knowledge. In this way, studying and training on donated human bodies can effectively help in reducing surgical complications, resulting in a better surgery for the patient and helping the clinician align with the concept of “hic mors gaudet succurrere vitae”.

## 5. Conclusions

Within the limitations of this study, the malar region offers adequate bone widths to place four zygomatic implants, two on each side, in the rehabilitation of the severely atrophic edentulous patient; however, this surgery still remains related to possible complications. Anatomical study combined with adequate training on a donated body is of fundamental importance and can allow the clinician to reduce the risks related to surgical complications and to intraoperatively identify a safe area for a correct zygomatic implant positioning.

## Figures and Tables

**Table 1:** Mean distances (mm), standard deviations (SD), and minimum and maximum values for malar bone measurements, including infraorbital foramen to pyriform nasal aperture (IF-PNA), infraorbital foramen to orbital floor (IF-OF), infraorbital foramen to zygomaticofacial foramen (IF-ZFF), zygomaticofacial foramen to orbital floor (ZFF-OF), height at point A (A-Height), distance from point A to point B (A-B), thickness at point A (A-Thickness), height at point B (B-Height), thickness at point B (B-Thickness), and depth of orbital floor (OFD).

	IF-PNA	IF-IM	IF-ZFF	ZFF-IM	A-Height	A-B	A-Thickness	B-Height	B-Thickness	OFD
Mean	18.66	7.65	26.72	7.43	14.02	19.02	3.58	6.3	2.88	5.74
SD	2.63	1.39	8.7	2.09	2.74	1.72	0.86	1.2	0.68	1.28
Minimum	9.5	4.3	14.6	3.6	9.3	15.3	1.6	4.3	1.3	3
Maximum	23.2	10.3	79.6	17.1	19.6	23.5	5.9	9.4	4.4	8

## Data Availability

The raw data supporting the conclusions of this article will be made available by the authors on request.
